# The application of extracorporeal membrane oxygenation in emergent airway management - a single-center retrospective study

**DOI:** 10.1186/s13019-024-02482-8

**Published:** 2024-01-23

**Authors:** Zhijun Fan, Simin Zhu, Jinling Chen, Junlin Wen, Binfei Li, Xiaozu Liao

**Affiliations:** Department of Anesthesiology, Zhongshan City People’s Hospital, East Sunwen road, Zhongshan, Guangdong Province 528403 China

**Keywords:** Emergent airway, Extracorporeal membrane oxygenation, Non-anticoagulation, Rapid-response team

## Abstract

**Background:**

Emergent airway occurrences pose a significant threat to patient life. Extracorporeal membrane oxygenation (ECMO) has been proven to be an effective method for managing emergent airways.

**Methods:**

A retrospective analysis was conducted on all patients receiving ECMO as an adjunct for emergent airway management from January 2018 to December 2022 at the People’s Hospital of Zhongshan City. We collected the basic information of the patients, their blood gas data before and after ECMO, the related parameters of ECMO, and the outcome and then analyzed and summarized these data.

**Results:**

Six patients, with an average age of 51.0(28–66) years, received veno-venous (VV)- ECMO as an adjunct due to emergent airway issues. The average ECMO support duration was 30.5(11–48) hours. All six patients were successfully weaned off ECMO support, with five (83.3%) being successfully discharged after a hospital stay of 15.5(7–55) days. All six patients underwent VV-ECMO through femoral-internal jugular vein cannulation. Among these, five patients, whose airway obstruction was due to hemorrhage, underwent a non-anticoagulant ECMO strategy with no recorded thrombotic events.

**Conclusions:**

The rapid establishment of ECMO support is aided by the establishment of a standardized ECMO initiation protocol and the formation of a multidisciplinary rapid-response ECMO team, which is particularly crucial for emergent airway management. When airway obstruction results from hemorrhagic factors, the early adoption of a non-anticoagulant ECMO strategy can be considered when implementing VV-ECMO.

## Background

An emergent airway refers to a situation where an airway obstruction arises due to various factors, leading to a significant compromise in patient ventilation and posing a direct threat to the patient’s life. In such instances, immediate intervention is required to establish effective ventilation. Techniques to enhance ventilation include the utilization of face masks, supraglottic airways, and attempts at tracheal intubation. Should these measures prove insufficient, the early initiation of emergent invasive airway management should be seriously considered. While most patients can effectively ameliorate their ventilation issues through this process, there are those who, due to factors such as extensive airway hemorrhage, cannot effectively restore ventilation via tracheal intubation. Such patients urgently require alternative strategies to swiftly rectify oxygenation problems.

Extracorporeal membrane oxygenation (ECMO) has been utilized to manage cardiac and respiratory failure for over four decades. In cases of respiratory failure, ECMO is broadly applied to patients with cardiopulmonary failure, including those suffering from acute respiratory distress syndrome (ARDS) and decompensated pulmonary arterial hypertension, as a bridging strategy before lung transplantation and for primary graft dysfunction post-lung transplantation [1]. Serving as a form of short-term cardiopulmonary support, ECMO aids in ensuring sufficient ventilation during surgical procedures. The American Society of Anesthesiologists (ASA) released an updated version of their practice guidelines for managing difficult airways in 2022, within which ECMO is recommended as a significant tool for emergent airway management [2]. Consequently, this study was conducted to retrospectively analyze and summarize the clinical experiences of ECMO application as an adjunctive treatment at the People’s Hospital of Zhongshan City for emergent airway management, with the aim of providing a reference for the management of patients with challenging emergent airways.

## Methods

This study was approved by the Ethics Committee of the People’s Hospital of Zhongshan City.

This study retrospectively analyzed all patients who received ECMO as an adjunct for emergent airway management at the People’s Hospital of Zhongshan City from January 2018 to December 2022. We collected the basic information of the patients, their blood gas data before and after ECMO, the related parameters of ECMO, and the prognosis. Data were collected from electronic medical records and are reported as median and min/max or frequencies and percentages.

### ECMO initiation

At the People’s Hospital of Zhongshan City, all ECMO procedures are performed by a multidisciplinary team comprising anesthesiologists, cardiothoracic surgeons, and vascular surgeons. This team has undergone standardized training and is in an emergency state 24 h a day, with a 5-minute in-hospital emergency response time.

### ECMO establishment and management

Veno-venous (VV) -ECMO was implemented for patients with severe respiratory failure but whose cardiac function had not yet failed due to hypoxia. Blood was drawn from the femoral vein, and oxygenated blood was returned via the internal jugular vein. The perfusion system consisted of a Medtronic Biopump centrifugal pump, a Medtronic oxygenator(Affinity NT), and a Carmeda heparin-coated ECMO kit provided by Medtronic, including Medtronic cannulation. Under ultrasound guidance, heparin-coated cannulas, A 19 Fr-21 Fr cannula was inserted into the femoral vein (The insertion depth was 35–45 cm) and A 15 Fr-17 Fr cannula was inserted into the right internal jugular vein (The insertion depth was 10–15 cm), respectively, were placed into the femoral vein and internal jugular vein. For patients with airway obstruction due to bleeding, a non-anticoagulant ECMO strategy was implemented. Those without bleeding risk were given a heparin load of 100 U/kg. Veno-arterial (VA)-ECMO was implemented for patients with severe cardiac failure or cardiac arrest due to hypoxia, with femoral arteriovenous cannulation chosen to establish ECMO. ECMO parameters were adjusted based on changes in the patient’s gas exchange, and the cause of the emergent airway was rapidly corrected with ECMO assistance. All patients were admitted to the ICU for monitoring. Once the factor causing the emergent airway was effectively managed and the patient’s respiration and circulation were stable without ECMO support, ECMO was withdrawn. Indications for VV-ECMO withdrawal were as follows: (1) Improvement in the patient’s original lung function and imaging. (2) Mechanical ventilation parameters: an inhaled oxygen concentration of < 50%, a tidal volume of 6–8 ml/kg, a peak airway pressure of < 30 cmH2O, a plateau airway pressure of < 25 cmH2O, and a positive end-expiratory pressure of ≤ 10 cmH2O, with satisfactory oxygenation maintained. (3) Arterial blood gas analysis: carbon dioxide elimination ability, oxygenation index, and internal environment stability.

### Statistical analysis

Data were collected from electronic medical records and are reported as median and min/max or frequencies and percentages.

## Results

**Fig. 1:**
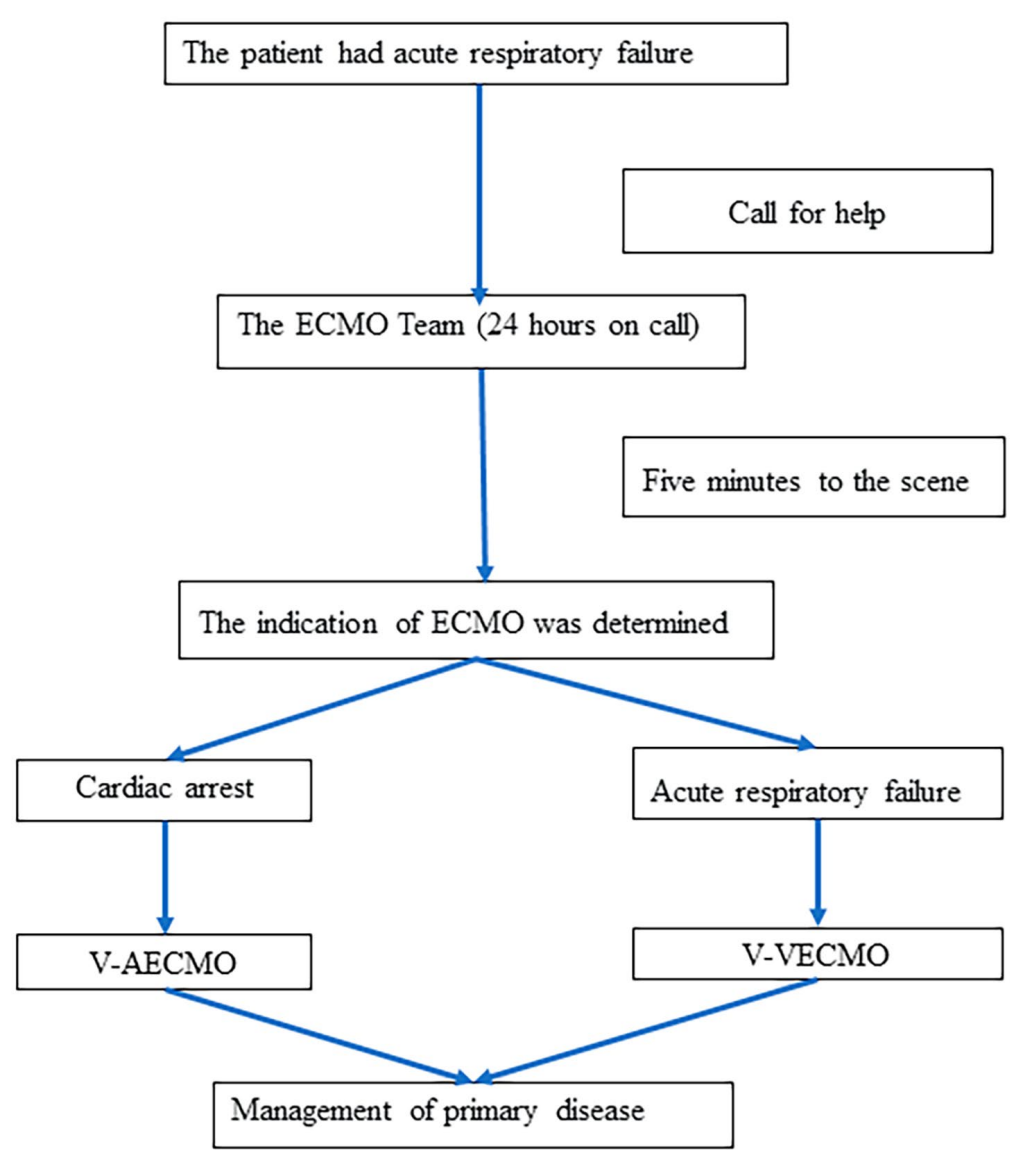
ECMO Rescue Initiation Process

A total of six patients with acute respiratory failure due to emergent airway issues were treated with ECMO following the initiation process at the People’s Hospital of Zhongshan City (Fig. 1).

All six patients received VV-ECMO assistance for emergent airways, including five males and one female, with an average age of 51.0(28–66) years. The ECMO assistance time for these patients was 30.5(11–48) hours. All six patients were successfully weaned from ECMO, and 5 (83.3%) were successfully discharged with a hospital stay of 15.5(7–55)days (Table 1).

**Table 1:** Baseline characteristics and outcomes

	Overall cohort (*n* = 6)
Age (years)	51.0(28–66)
Sex (Male/Female)	5/1
Body surface area (m^2^)	1.7(1.56–1.98)
Chronic lung disease (n (%))	1(16.6)
Weaning from ECMO (n (%))	6(100)
ECMO duration (h)	30.5(11–48)
Length of hospitalization (days)	15.5(7–55)
Hospital survival((n(%))	5(83.3)

**Fig. 2:**
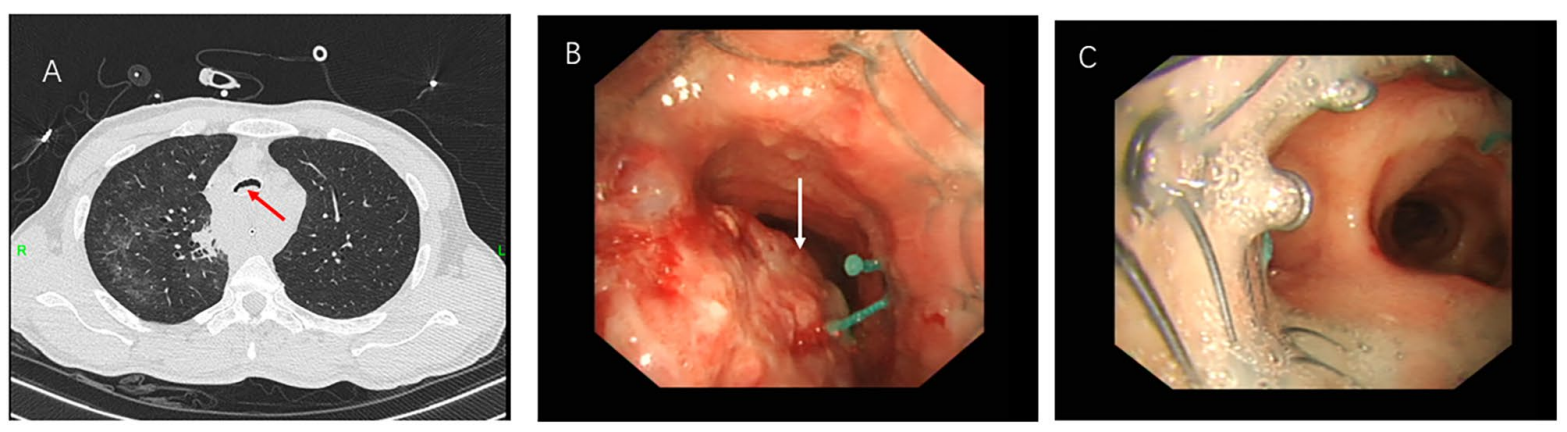
A 55-year-old male patient underwent airway stent implantation due to esophageal cancer invading the airway. (**A**: The main airway is invaded by the tumor, causing airway narrowing. **B**: The tumor invaded the inner airway, causing luminal narrowing. **C**: Under ECMO support, the airway stent is successfully adjusted, and the narrowing is relieved. The arrow points to the tumor inside the airway.)

**Fig. 3:**
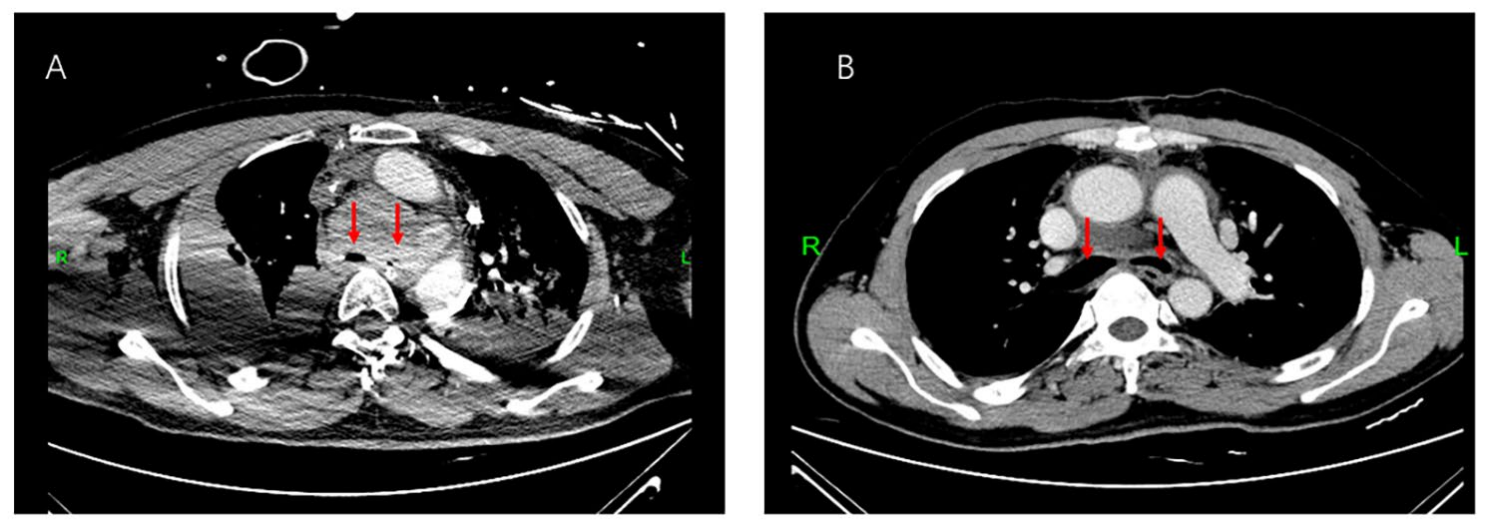
A 46-year-old male patient with esophageal cancer and esophageal stent implantation, with the tumor compressing the airway. (**A**: The airway near the tracheal prominence is compressed and significantly narrowed. **B**: After placement of the tracheal stent, the narrowing is relieved. **C**: Mucosal congestion and bleeding. **D**: Airway bleeding during the stent placement process. The arrow points to the airway and congested mucosa.)

**Fig. 4:**
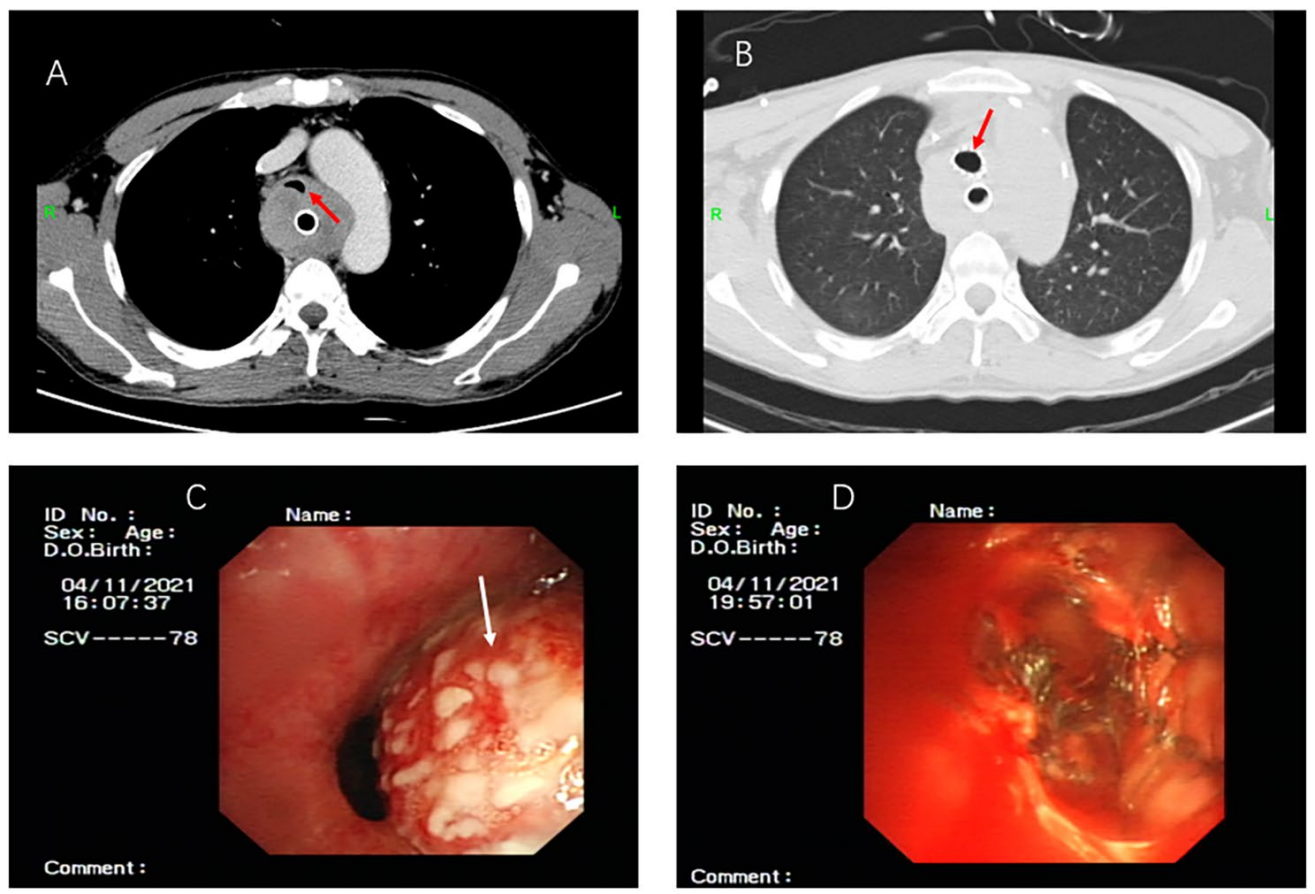
A 47-year-old male patient with a neck artery rupture causing bleeding and compressing the airway. (**A**: A cervical hematoma compressing the left and right bronchi. **B**: After surgery, compression of the left and right bronchi is relieved. The arrow points to the left and right bronchi.)

**Fig. 5:**
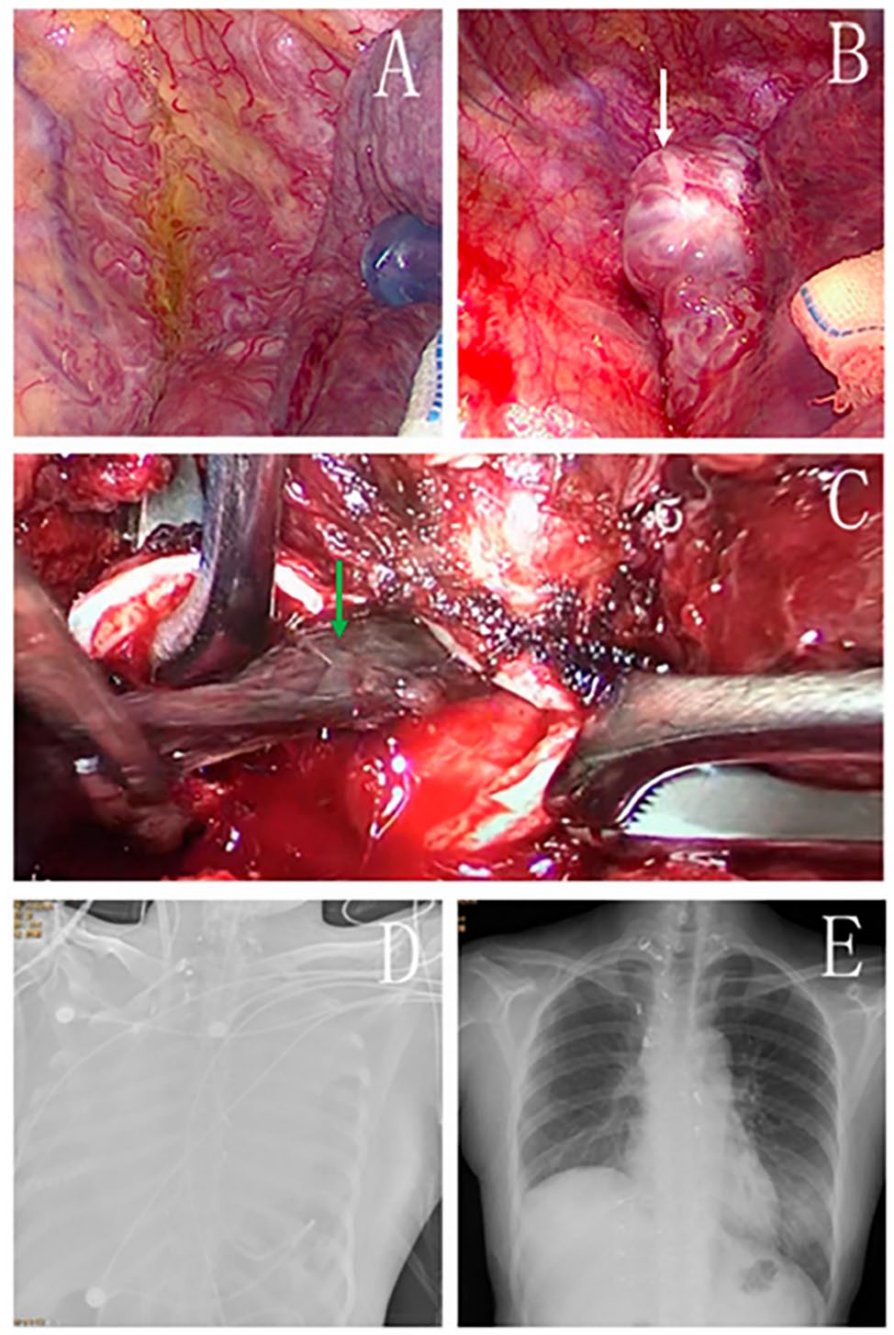
A 28-year-old female suffered from airway obstruction due to the rupture and bleeding of a tracheal vascular tumor. (**A**. Massive abnormal vascular hyperplasia in the right upper mediastinum. The normal mediastinum pleura was not visualized. **B**. The hemangioma was composed of a large number of criss-cross arteriovenous masses. **C**. Intraoperatively, the right intermediate bronchus was opened. Several blood clots can be seen blocking the bronchus. The blood clots in the main airway were completely removed. **D**. Chest X-ray after hemoptysis worsened. **E**. Chest X-ray at discharge.)

The reasons for ECMO assistance included four cases due to massive airway bleeding leading to airway obstruction, among which two were due to incomplete airway obstruction caused by bleeding during the adjustment of the tracheal stent (Figs. 2 and 3), one due to airway compression caused by bleeding from a ruptured anomalous aortic arch (Fig. 4), and one due to incomplete airway obstruction caused by bleeding from a ruptured bronchial angioma (Fig. 5). One case was due to a widespread and persistent airway spasm during an asthma attack. VV-ECMO was established through the femoral vein-internal jugular vein in all six patients. The five patients with airway obstruction due to bleeding used a non-anticoagulant ECMO strategy, and none experienced thrombotic events (Table 2).

**Table 2:** Baseline characteristics, details of early ECMO and outcomes

Case number	Case1	Case2	Case3	Case4	Case5	Case6
Age (years)	55	47	46	56	66	28
Sex	Male	Male	Male	Male	Male	Female
Body surface area (m^2^)	1.65	1.98	1.56	1.92	1.88	1.71
Causes of Airway obstruction	Airway compression by tumor + airway hemorrhage	Arterial bleeding compresses the airway	Airway compression by tumor + airway hemorrhage	Severe acute intractable asthma	Pulmonary hemorrhage due to aspergillus infection	The tracheal hemangioma was ruptured and bleeding
ECMO mode	VV	VV	VV	VV	VV	VV
Configuration	Fem-IJ	Fem-IJ	Fem-IJ	Fem-IJ	Fem-IJ	Fem-IJ
Cannula size (French) (drainage/re-infusion)	21/17	21/17	21/17	21/17	21/17	21/17
ECMO blood flow (l/min)	3.5	3.6	3.4	3.5	3.1	3.6
Bolus UFH (U)	0	0	0	0	100	0
Maintenance UFH (U/h)	0	0	0	250	0	0
APTT during ECMO(lowest, highest)(s)	46.2,49.5	33.3,38.5	29.2,30.7	55.6,66.8	24.8,34.5	29.2,33.3
Thrombotic events(Yes/No)	No	No	No	No	No	No
ECMO duration (h)	12	25	36	11	48	36
Intervention of airway obstruction after ECMO	tracheal stent + hemostasis	Surgery	tracheal stent + hemostasis	Glucocorticoids, β2 -agonists	pulmonary lobectomy	pulmonary lobectomy
Weaning from ECMO(Yes/No)	Yes	Yes	Yes	Yes	Yes	Yes
Length of hospitalization (days)	34	18	7	11	13	55
Hospital survival(Yes/No)	Yes	Yes	Yes	Yes	No	Yes

Fem-IJ: femoral vein-internal jugular vein

Six patients suffered from acute respiratory failure after emergency airway. After ECMO assisted treatment, acute respiratory failure was effectively treated, and oxygenation and internal environment of patients were effectively improved (Table 3).

**Table 3:** Blood gas analysis before and after ECMO

	Reference value	Time	Case1	Case2	Case3	Case4	Case5	Case6
PH value	7.35–7.45	Pre-ECMO	7.12	7.2	7.1	7.01	7.55	7.26
		Post-ECMO	7.45	7.38	7.52	7.36	7.39	7.45
PaCO_2_	35–48 mmHg	Pre-ECMO	125	106	> 130	90	100	98
		Post-ECMO	35	39.6	35.2	36.9	32	35
PaO_2_	83–108 mmHg	Pre-ECMO	50	48	56	59	45	52
		Post-ECMO	191	80.2	239	245	120	156

## Discussion

This study suggests that ECMO is beneficial in ensuring ventilation functions (oxygenation and CO2 clearance) for patients suffering from respiratory failure due to various causes of airway obstruction. Under the support of ECMO, medical personnel have relatively enough time to plan and implement the management of the patient’s primary disease.

In this study, patients were able to receive ECMO support in a timely manner after an emergency airway event, which may be related to the establishment of a hospital-wide ECMO initiation process and a multidisciplinary ECMO rapid response team in our center. Previous studies have shown that the establishment of a rapid response team to manage pulmonary embolism patients significantly increases the success rate of patient rescue [3]. Another study suggested that a fast ECMO response team can reduce neurological complications in ECPR [4]. Although the speed of emergency airway ECMO establishment can be improved by a rapid ECMO response team, the rescue of emergency airway patients is still challenging. Failure to quickly correct and improve ventilation in an emergency airway may result in severe hypoxia in the patient, affecting the patient’s prognosis. It is crucial to prevent the occurrence of emergency airways; therefore, it may be a good choice to adopt preventive VV-ECMO for surgeries involving high-risk airway interventions, providing adequate safety assurance to address airway issues [5, 6]. In this study, six patients initiated ECMO in a timely manner without cardiac arrest. All used the VV-ECMO mode, and the prognosis was good. For patients who have cardiac arrest and need to initiate ECPR [7], the mortality rate of patients undergoing ECPR is high, with a survival discharge rate of approximately 25% [8]. Neurological prognosis is related to the time of ECMO initiation, and the duration of the patient’s low-flow time significantly affects the neurological prognosis [9].

In this study, the ECMO assistance time was 30.5 (11.7–39.0) hours, the ECMO weaning rate was 100%, and the survival discharge rate (83.3%) was significantly higher than that of patients with severe pneumonia undergoing VV-ECMO (54%) [10]. The duration of ECMO support mainly depends on the correction of the primary disease. The primary diseases of the cases in this study were non-pulmonary factors leading to respiratory failure, so as long as the factors causing the difficulty of ventilation are handled, the patient’s respiratory function can recover in a short time.

In this study, the primary disease of five patients was bleeding caused by various factors leading to airway blockage. Hemostasis is key; therefore, the five patients in this group adopted a non-anticoagulation VV-ECMO strategy. Five patients quickly corrected the cause of bleeding through methods such as transfusion of coagulation substances, electrocoagulation hemostasis, and surgical operation hemostasis. With the continuous development of ECMO equipment (i.e., heparin-coated cannulas), short-term anticoagulation (heparin push) during the ECMO process can be reduced or even cancelled, thus enhancing the safety of invasive procedures during ECMO. Implementing anticoagulation in VV-ECMO support with invasive operations will increase the risk of bleeding [11]. There is much research proving the safety of non-anticoagulation ECMO during VV-ECMO support [12]. One study suggested that in ECMO, the absence of routine systemic anticoagulation is not related to higher mortality, centrifugal pump dysfunction, or thrombus formation complications, and the requirement for blood product transfusion is lower for non-anticoagulation ECMO patients [13]. In this study, no thrombotic events occurred in the group of patients implementing the non-anticoagulation strategy, which may be related to the short ECMO support time and relatively high ECMO flow. Although the non-anticoagulation ECMO strategy has been successful in many studies, thrombotic events are still a factor affecting the prognosis of ECMO patients. Thrombotic events are very common during VV-ECMO and have a strong cumulative correlation with in-hospital mortality. Although thrombotic events are more common, the risk of death due to bleeding in hospitalized patients is higher, so a tailored VV-ECMO anticoagulation management method may be adopted [14]. The criteria for adopting a non-anticoagulation ECMO strategy for VV-ECMO patients with airway obstruction due to hemorrhagic factors may be as follows: there are factors with a high risk of bleeding, such as the implementation of surgical operations, the expected ECMO support time is short, maintaining a relatively high flow, and strengthening thrombotic event monitoring.

In this study, one patient was assisted by ECMO due to acute respiratory failure caused by persistent asthma, effectively reducing lung injury caused by the ventilator. A data study from the multicenter Extracorporeal Life Support Organization (ELSO) registration system shows that compared with ECMO support for other respiratory diseases, the survival rate of asthma patients with ECMO support is higher [15].

The main limitations of this study are the single-center research and small sample size, using retrospective, non-random, and non-controlled methods. However, conducting random, controlled, and/or double-blind studies in patients in life-threatening clinical states is impossible and unethical. Although this study successfully used the non-anticoagulation ECMO strategy, more research is needed to confirm its safety, especially how to implement the non-anticoagulation ECMO strategy, such as how long it can be used, and how to monitor it more precisely: D-dimer, thromboelastogram, etc.

## Conclusions

The establishment of a standardized ECMO initiation process and a multidisciplinary rapid response ECMO team is beneficial to the rapid establishment of ECMO support, especially in the field of emergency airways. For VV-ECMO caused by hemorrhagic factors leading to airway obstruction, an early non-anticoagulation ECMO strategy can be adopted. When patients have an emergency airway, ECMO should be initiated early for treatment, thus reducing the occurrence of ECPR and improving patient prognosis.

## Abbreviations

ECMO: Extracorporeal membrane oxygenation
ARDS: Acute respiratory distress syndrome
ASA: American Society of Anesthesiologists
VV: Veno-venous
VA-ECMO: Veno-arterial ECMO
ECPR: Extracorporeal cardiopulmonary resuscitation
Fem-IJ: Femoral vein-internal jugular vein
